# Dangers from Decaying Teeth

**Published:** 1881-12

**Authors:** 


### DANGERS FROM DECAYING TEETH.

IT is not true that savage tribes and semi-civilized people have sounder teeth, as a rule, than are found among the highly civilized. On the contrary, the conditions of culture and refinement favor the preservation of health through care and cleanliness. Cleopatra was said to have been the most beautiful woman of her time; but what would be thought of the claims of a modern belle whose smiles should be marred by a display of teeth in various stages of decay, as were those of Egypt's famous queen.

Beautiful and well-kept teeth do more than anything else to atone for defective features. But there are other considerations of far greater importance than mere personal beauty, which require that the teeth should be carefully watched from infancy to old age.

Neglected teeth are frequently the cause of disease and death. I do not remember ever to have seen a case of facial neuralgia that could not be traced to this cause. In recent cases a cure can generally be effected by extracting such teeth as are much decayed and filling those that can be saved in that way. I have recently had two patients, one of whom had suffered almost constantly for two years with neuralgia of the face and neck. I advised the removal of two teeth and the filling of all others where cavities could be found; and since that time, about two months, there has been no return of the trouble. The other, a young lady of sixteen, with teeth that are beautiful and perfect in appearance, has suffered frequently with neuralgic headache. I could discover no defect in the teeth and began to distrust my theory of the origin of neuralgia in the head; but I advised her to consult a dentist, and he discovered narrow but deep cavities in the crowns of five teeth; these were filled a month ago, and there has been no return of headache. It should be known that ordinarily cures are thus effected in recent cases only. I have in several cases of long standing advised extraction of all the teeth. There is an interruption of the disease usually for a few months, but it is apt to return again, but with diminished force.

Decay is death, and dead and decaying teeth poison the blood by absorption and debiltate the patient. There is nothing so repulsive to the sense of smell as a decayed tooth; nothing that more surely carries disease to the vital organs than to inhale effluvia from such sources of corruption ; nothing that so nauseates and disgusts everybody as fetid breath emanating from such decay.

I never saw an adult person with bad teeth who had not dyspepsia, and who was not more or less untidy. Almost unconsciously, there is a want of self respect. If cleanliness is next to godliness, why should not uncleanliness be next to something else ?

Parents should look often and carefully to their children's teeth, as well as to their own. If decay commences, even with the first teeth, they should be filled. If the permanent front teeth project outward so as to prevent the lips from closing easily, the defect should be remedied at all hazards, for if the air habitually reaches the lungs through the mouth, bronchial trouble is certain to result sooner or later.

Parasites always infest neglected teeth ; and tartar accumulates around them and burrows downward, destroying the periostium and the teeth. To guard against these evils, great care is necessary. Soap will destroy the parasites, and proper tooth powder, used occasionally, will prevent the tartar from accumulating. I would advise the greatest caution in the selection of preparations for the teeth. Many of those that are advertised contain acids, and although they clean the teeth perfectly, at first, they will in time inevitably destroy them. The only one that I have found which seems to combine the essentials of a complete and safe preparation for the the teeth is "Sozodont." It matters not, however, what plan is adopted to preserve the teeth, so long as the object is attained and the methods employed are not destructive.

